# Early-stage lung cancer associated with higher frequency of chest x-ray up to three years prior to diagnosis

**DOI:** 10.1017/S1463423622000573

**Published:** 2022-11-02

**Authors:** Stephen H. Bradley, Martyn P.T. Kennedy, Matthew E.J. Callister

**Affiliations:** 1 National Institute of Health Research Academic Clinical Fellow, Academic Unit of Primary Care, University of Leeds, Leeds, UK; 2 Consultant Respiratory Physician, Leeds Teaching Hospitals NHS Trust, Department of Respiratory Medicine, St James University Hospital, Leeds, UK

**Keywords:** cancer diagnosis, chest x-ray, lung cancer, radiography

## Abstract

**Objectives:**

Symptom awareness campaigns have contributed to improved early detection of lung cancer. Previous research suggests that this may have been achieved partly by diagnosing lung cancer in those who were not experiencing symptoms of their cancer. This study aimed to explore the relationship between frequency of chest x-ray in the three years prior to diagnosis and stage at diagnosis.

**Settings:**

Lung cancer service in a UK teaching hospital.

**Participants:**

Patients diagnosed with lung cancer between 2010 and 2013 were identified. The number of chest x-rays for each patient in the three years prior to diagnosis was recorded. Statistical analysis of chest x-ray frequency comparing patients with early- and late-stage disease was performed.

**Results:**

One-thousand seven-hundred fifty patients were included – 589 (33.7%) with stage I/II and 1,161 (66.3%) with stage III/IV disease. All patients had at least one chest x-ray in the six months prior to diagnosis. Those with early-stage disease had more chest x-rays in this period (1.32 vs 1.15 radiographs per patient, *P* = 0.009). In the period 36 months to six months prior to lung cancer diagnosis, this disparity was even greater (1.70 vs 0.92, radiographs per patient, *P* < 0.001).

**Conclusions:**

Increased rates of chest x-ray are likely to contribute to earlier detection. Given the known symptom lead time many patients diagnosed through chest x-ray may not have been experiencing symptoms caused by their cancer. The number of chest x-rays performed could reflect patient and/or clinician behaviours in response to symptoms.

## Background

Poor lung cancer outcomes in countries such as the UK, relative to similar settings, have been attributed in part to a later stage at diagnosis (Walters *et al.*, 2013). This may be due to the more conservative use of CT imaging compared to international peers (Eurostat, 2019), which may in turn lead to fewer incidentally diagnosed lung cancers. Although little is known about variation in rates of referral for chest x-ray specifically, individual GPs are known to exhibit varying propensities to arrange diagnostic tests (Verstappen *et al.*, 2004), while patients present with varying promptness following the onset of symptoms (patient interval) (Keeble *et al.*, 2014).

Improved survival is strongly associated with diagnosis in earlier stages of lung cancer (Cancer Research UK). Achieving earlier stage of diagnosis is a major policy focus in the UK with England’s National Health Service (NHS) aiming to achieve diagnosis of 75% of all cancers in either stage I or II by 2028. (NHS England)

Although annual screening with chest x-ray has failed to achieve reduced mortality (Oken *et al.*, 2011), symptom awareness campaigns have succeeded in increasing the uptake of chest x-ray, demonstrated greater proportions diagnosed at earlier stages and improved survival (Kennedy *et al.*, 2018; Peake, 2018). Case-control evidence has shown that almost all of the increased presentations to health services amongst those with lung cancer, compared to controls, occur in the six months prior to diagnosis (Ades *et al.*, 2014). In the two years prior to diagnosis of lung cancer, 27–48% of symptoms which could justify referral for chest x-ray are not actually symptoms *caused* by the lung cancer (Biswas *et al.*, 2014). This suggests that patients that stand to benefit the most through early diagnosis initiatives are those for whom their symptoms were often not attributable to the cancer. The effect of symptom awareness campaigns may therefore be partly due to increased serendipitous detection through promoting a permissive attitude towards imaging of symptomatic patients by GPs. If this is the case, we might expect that patients who are diagnosed with earlier stage disease are also those who are exposed to increased rates of chest x-ray in the years prior to diagnosis, long before the median symptom lead time of three months (Ades *et al.*, 2014). The authors are not aware of any published studies which examine the frequency of imaging by stage of lung cancer at diagnosis. This study aims to analyse the frequency of chest radiographs prior to the development of lung cancer symptoms according to stage at diagnosis.

## Methods

Clinical data, including age, stage of lung cancer at diagnosis, lung cancer histology and one-year survival, were collected retrospectively from a database that includes all patients diagnosed with lung cancer in Leeds Teaching Hospitals Trust, between June 2010 and December 2013. Patients with small cell lung cancer were excluded due to the different clinical presentation and natural history of this disease.

The dates of all chest radiographs performed prior to a diagnosis of lung cancer were collected retrospectively from local radiology department data. These included both community and hospital-requested radiographs performed at any of the Leeds Teaching Hospital Trust sites.

These data were linked at an individual patient level by NHS number. Day zero was set as the date on which the patient was first seen by a member of the lung cancer team. The number of chest radiographs performed in the three years prior to this date was recorded in six-month periods.

Statistical analysis of chest radiograph frequency comparing patients who were diagnosed with stage I or II disease (‘early-stage’) with those diagnosed with stage III or IV stage (‘late-stage’) disease was performed using Mann-Whitney U test with normal approximation. The 7^th^ edition TNM staging for lung cancer was used, as this was the staging system in use at that time.

## Results

There were 1,750 patients included in the study, of whom 589 (33.7%) had early-stage (I or II) and 1,161 (66.3%) had late-stage (III or IV) disease. Table 1 describes the patient characteristics of these groups. One-year overall survival for all patients was 42.2%.

**Table 1.** Summary of study population

	Stage I or II *n* = 589	Stage III or IV *n* = 1,161	*P*-value
**Age (mean, SD)**	73.6 ± 10.1 years	72.6 ± 11.2 years	0.068
**WHO Performance Status 0–2**	441, 74.9%	684, 58.9%	<0.001
**WHO Performance Status 3–4**	148, 25.1%	477, 41.1%	
**Route to diagnosis:**			
**Emergency admission**	65, 11.0%	422, 36.3%	<0.001
**Outpatient**	524, 89.0%	739, 63.7%	
**Pathological subtype:**			
**Adenocarcinoma**	201, 34.1%	332, 28.6%	
**Large cell**	16, 2.7%	45, 3.9%	
**Squamous cell**	164, 27.8%	282, 24.2%	
**NOS**	20, 3.4%	139, 12.0%	
**Carcinoid**	13, 2.2%	3, 0.3%	
**Unknown**	175, 29.7%	360, 31.0%	
**One-year survival with 95% CI**	76.8%, 73.4–80.3%	24.7%, 22.4–27.3%	<0.001

All patients had at least one chest x-ray performed in the six months prior to diagnosis. Those subsequently diagnosed with early-stage lung cancer, however, had significantly more chest radiographs than those with late-stage disease in this period (1.32 vs 1.15 radiographs per patient, *P* = 0.009).

**Figure 1.**
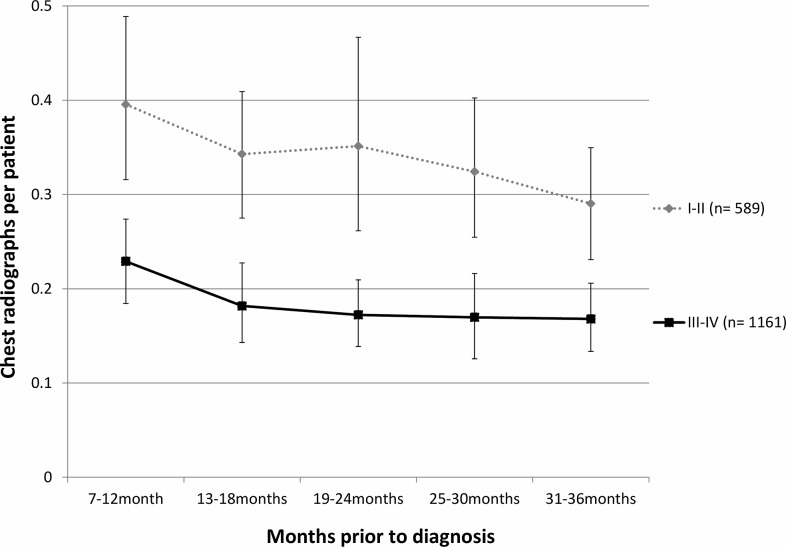
Chest radiographs per patient in 6-month periods prior to diagnosis

The frequency of chest radiographs per patient per six-month period during the three years prior to lung cancer diagnosis, excluding the final 6 months, is shown in Figure 1 according to stage at diagnosis. Patients subsequently diagnosed with early-stage cancer had significantly more chest radiographs performed during this period compared to late-stage patients (1.70 vs 0.92, *P* < 0.001).

The proportion of patients with no chest radiograph in the three years before diagnosis, excluding the six months immediately before diagnosis, was higher in late-stage group than in those with early-stage disease (64.6% vs 45.0%, *P* < 0.001).

## Strengths & limitations

This is the only study which has examined the numbers of chest x-rays performed for patients in the years prior to diagnosis, stratified by early- and late-stage diagnosis. Demographic factors, such as socio-economic status, which may have had acted as a confounder with both chest x-ray status and stage at diagnosis, were not taken into account. However, this is unlikely to significantly alter interpretation of the results since socio-economic status is not associated with stage of disease at diagnosis (Cheyne *et al.*, 2013; Lyratzopoulos *et al.*, 2013). Adjustment was not performed for age or sex. While female sex and younger age are associated with a greater risk of late-stage diagnosis, it seems likely that this effect is attributable to the lower suspicion for cancer in younger and female patients which would be reflected by the number of chest x-rays performed for these patients (Lyratzopoulos *et al.*, 2013).

The study findings could have been influenced by over-diagnosis, with incidental detection of more indolent early-stage cancers in those having more frequent chest radiographs, which would have otherwise gone undetected.

## Discussion

This study has demonstrated that patients that are subsequently diagnosed with early-stage lung cancer had more frequent chest radiographs at all points in the three years prior to diagnosis. Patients who are at risk of lung cancer are also more likely to experience other respiratory disorders and symptoms (Iyen-Omofoman *et al.*, 2013). Given that the median symptom lead time for lung cancer has been estimated to be under three months (Ades *et al.*, 2014), it seems likely that many of the early-stage cancers were detected because of investigation initiated for symptoms not caused by their lung cancer. The number of chest x-rays performed is likely to be influenced by individual patient and GP factors, such as the likelihood of individual patients to present to their GP when they have symptoms, the propensity of their GP to investigate these symptoms with chest x-ray and patients’ own preference for investigation chest x-ray.

Patients who were more likely to be investigated with chest x-ray, reflected in higher chest x-ray rates, may have been more likely to have experienced such serendipitous, early detection. This corroborates the insight from case-control studies which has suggested that a large proportion of symptomatic patients in whom early-stage lung cancer was detected did not have symptoms caused by cancer (Biswas *et al.*, 2014).

These insights help explain how policies which increase uptake of chest x-ray achieve earlier diagnosis, and how that effect may be maintained and enhanced. While previous screening trial evidence suggests that a uniform policy of annual screening with chest x-ray does not achieve improved survival (Oken *et al.*, 2011), a pragmatic approach to the promotion of opportunistic imaging does appear to yield benefits, perhaps modulated by clinicians’ and patients’ intuition of those at great risk.

In England between 2011 and 2014, the ‘be clear on cancer’ campaign targeted both GPs and patients, promoting investigation of respiratory symptoms lasting three weeks or more in patients at risk of lung cancer with chest x-ray (Peake, 2018). The largest increases in imaging took place in response to the most intensive campaign activity, and it is possible that sustaining increases in symptomatic patient presentations and referral by clinicians requires ongoing engagement. In Leeds, a symptom awareness campaign coupled with a coordinated approach across primary and secondary care to increase chest x-ray rates in symptomatic patients was associated with a ‘stage shift’ in diagnosis towards more early-stage disease (Kennedy *et al.*, 2018). Other research has shown that there may be benefits in targeting patients at high risk of lung cancer with information encouraging them to present should they develop symptoms (Emery *et al.*, 2019).

The present study suggests that those patients who tend to be investigated more frequently with chest x-ray, perhaps because they are more inclined to present when symptomatic, because their GPs are more inclined to investigate with chest x-ray, or both, are more likely to benefit from discovery of lung cancer at an early stage. Patients registered at GP practices that make greater use of urgent suspected cancer pathways are associated with favourable cancer outcomes (Round *et al.*, 2020). Since almost all primary care criteria for such referrals are based on imaging findings (NICE, 2015), it is plausible that propensity to investigate with chest x-ray is also associated with improved outcomes. Continued efforts to encourage patients to present when they have symptoms and for GPs to investigate with chest x-ray when they attend may well be justified, even if the likelihood that symptoms are being caused by lung cancer on any one attendance is low.

Given the complex interplay of individual and system factors which influence both patients’ propensity to present and clinicians’ propensity to request imaging, additional research which takes account of patient and clinician factors would help clarify the reasons for the differentials in imaging rates between patients with early-stage and late-stage disease. In order to determine whether encouraging GPs to adopt a lower threshold for imaging would be useful research could also explore whether patients who are diagnosed with lung cancer in general practices which undertake greater numbers of chest x-rays in their patient populations, following appropriate adjustment for confounders such as smoking prevalence, age and deprivation, are diagnosed at earlier stages of disease compared to practices which request fewer chest x-rays.
